# Leucine and Glutamic Acid as a Biomarker of Sarcopenic Risk in Japanese People with Type 2 Diabetes

**DOI:** 10.3390/nu15102400

**Published:** 2023-05-21

**Authors:** Hanako Nakajima, Hiroshi Okada, Ayaka Kobayashi, Fuyuko Takahashi, Takuro Okamura, Yoshitaka Hashimoto, Naoko Nakanishi, Takafumi Senmaru, Emi Ushigome, Masahide Hamaguchi, Michiaki Fukui

**Affiliations:** 1 Department of Endocrinology and Metabolism, Graduate School of Medical Science, Kyoto Prefectural University of Medicine, Kyoto 602-0841, Japan; tabahana@koto.kpu-m.ac.jp (H.N.);; 2 Nutrition Division, Saiseikai Suita Hospital, Osaka 564-0013, Japan; 3 Department of Diabetes and Endocrinology, Matsushita Memorial Hospital, Osaka 570-8540, Japan

**Keywords:** metabolomics analysis, leucine, glutamic acid, type 2 diabetes, sarcopenia

## Abstract

This study aimed to identify the serum metabolites associated with sarcopenic risk in Japanese patients with type 2 diabetes, determine the effect of dietary protein intake on the serum metabolic profile, and examine its association with sarcopenia. Ninety-nine Japanese patients with type 2 diabetes were included, and sarcopenic risk was defined as low muscle mass or strength. Seventeen serum metabolites were quantified after gas chromatography–mass spectrometry analysis. The relationship between dietary protein intake and the metabolites concerning sarcopenia was analyzed, and the factors affecting sarcopenic risk were clarified. Twenty-seven patients were classified as being at risk of sarcopenia, the same as the general risk, which was associated with older age, a longer duration of the disease, and a lower body mass index. Low levels of leucine and glutamic acid were significantly associated with low muscle strength (*p* = 0.002 and *p* < 0.001, respectively), and leucine was also associated with muscle mass (*p* = 0.001). Lower levels of glutamic acid had higher odds of sarcopenic risk after being adjusted for age and HbA1c (adjusted OR 4.27, 95% CI 1.07–17.11, *p* = 0.041), but not for leucine. Leucine and glutamic acid can serve as useful biomarkers for sarcopenia, highlighting potential targets for its prevention.

## 1. Introduction

Sarcopenia is one of the most critical complications for patients with type 2 diabetes (T2D), which can directly affect prognosis and significantly reduce their quality of life, including the risk of bone fractures and frailty. The amount of people with diabetes has been increasing rapidly, with estimates claiming that by 2045 there will be over 700 million patients worldwide [1]. People with T2D are known to suffer from insulin resistance, inflammation, AGE accumulation, and oxidative stress that affect muscle mass and strength, systemic metabolism, and mitochondrial dysfunction, resulting in sarcopenia [2,3,4]. It has been reported that even patients with type 1 diabetes can develop sarcopenia as a long-term complication [5]. Additionally, in recent years, large-scale clinical trials have reported the usefulness of SGLT2 for cardiovascular disease, and the guidelines recommend the drugs for diabetes, but some reports suggest the possibility of the progression of sarcopenia or loss of muscle mass, based on the mechanism of pharmacological effects [1]. Preventing sarcopenia is crucial, especially for east Asian patients with diabetes, who often have less muscle mass than Western people. According to systematic reviews and randomized control trials, nutritional therapy, including branched-chain amino acid (BCAA) leucine and protein supplementation with whey protein, has been shown to have benefits in the treatment of sarcopenia, leading to increases in the muscle protein synthesis rate, total body muscle mass, and lean muscle mass [6,7,8,9].

Proteins are digested and metabolized into amino acids and other metabolites, which are then absorbed through the gut and transported in the blood plasma [10]. However, since dietary habits vary across countries, it is important to investigate whether dietary composition affects the sarcopenic risk in Japanese patients. Metabolome analysis has been increasingly used recently with the advances in technology, which has enabled the comprehensive quantification of the metabolites in various materials, including not only serum but also organs and feces, and is valuable to the investigation of novel metabolic pathways or substance interactions. Additionally, this form of analysis can comprehensively analyze serum metabolites after absorption, but no reports have quantitatively evaluated and compared metabolite concentrations rather than their relative ratios [11,12]. Several previous reports have examined sarcopenia cross-sectionally using metabolome analysis, body composition, and hand grip strength (GS). However, there have been no reports on sarcopenia in Japanese patients with T2D.

To address this gap in research on sarcopenia in Japanese patients with T2D, the present study focuses on the association between dietary nutrients ingested and serum metabolites and their impact on the risk of developing low muscle strength, low muscle mass, or sarcopenia. Specifically, this study aims to explore a dietary approach that could reduce the sarcopenic risk.

## 2. Materials and Methods

### 2.1. Study Population

The present study was a sub-analysis of the KAMOGAWA-DM cohort study, which is a large-scale, prospective cohort study aimed at investigating the clinical course and risk factors for diabetic complications in Japan (approval number: RBMR-E-466-6); its details are described elsewhere [13]. For the present study, we included patients with complete questionnaire data from January 2016 to December 2018. Patients without T2D, those with no data from the multifrequency impedance body composition analyzer and GS, whose questionnaires were incomplete, or those who had their serum samples stored were excluded from the study. Considering the possibility that serum metabolites may have different effects on sarcopenic risk depending on the individual’s age, subgroup analyses were performed only for patients aged ≥ 65 years old. This study was approved by the local research ethics committee and carried out in accordance with the Declaration of Helsinki. All patients provided written informed consent.

### 2.2. Data Collection

The weight and height of the patients were measured using an automatic weight and height meter, while the duration of the T2D was assessed through a standardized questionnaire. Data on diabetic medication, including the SGLT2 inhibitor and insulin, which may affect sarcopenia, were obtained from the subject’s medical record. We assessed their exercise habits through a standardized questionnaire. We defined a regular exerciser as someone that regularly played some kind of sport at least once a week. Blood samples were collected from patients after an overnight fast, and their fasting plasma glucose, the levels of glycated hemoglobin (HbA1c), and creatinine were measured in their serum.

### 2.3. Definition of Sarcopenic Risk

Data on body weight (kg), appendicular muscle mass (kg), and body fat mass (kg) were collected using a multifrequency impedance body composition analyzer [14]. Body mass index (BMI, kg/m^2^) was calculated by dividing the body weight (kg) by the square of the height (m) and ideal body weight, namely 22 multiplied by the square of the patient’s height (m) [15]. The skeletal muscle mass index (SMI, kg/m^2^), calculated as the appendicular muscle mass divided by the square of the height (m) [16], was also determined.

We measured the GS twice on both hands using a hand grip dynamometer (Smedley; Takei Scientific Instruments, Niigata, Japan). According to the updated version of the consensus report by the Asian Working Group for Sarcopenia [17], an SMI < 7 kg/m^2^ in males and <5.4 kg/m^2^ in females, and a GS < 28 kg in males and <18 kg in females are defined as sarcopenia. Because of the small number of patients who had both a low GS and a low SMI in this study, we defined sarcopenic risk as patients who had a low SMI or a low GS, indicating low muscle mass or strength.

### 2.4. Metabolomic Analyses

Plasma samples were stored at −80 °C until further use. The amino acids and organic acids in the serum were analyzed using gas chromatography–mass spectrometry (GC-MS) with an Agilent 7890B/7000D system (Agilent Technologies, Santa Clara, CA, USA). Briefly, the serum samples (50 µL) were added to 800 μL of acetonitrile and 150 μL of diluted water and shaken at 1000 rpm for three minutes at 37 °C. The samples were then centrifuged at 14,000× *g* rpm for three minutes at room temperature, and the resulting supernatant (500 µL) was separated and added to 500 μL of acetonitrile. The pH was then adjusted to 8 using 0.2 mol/L NaOH.

The amino acid and organic acid concentrations were determined using the on-line solid phase extraction (SPE) method with GC-MS. The SPE-GC system SGI-M100 (AiSTI SCIENCE, Wakayama, Japan) automatically performed the SPE and injection into the GC-MS system after the sample was added to the vial. Flash-SPE ACXs (AiSTI SCIENCE) were used for solid stratification. To measure the levels of amino acids and organic acids, 50 µL aliquots of each sample extract were loaded onto the solid phase, washed with acetonitrile and water (1:1), dehydrated with acetonitrile, and impregnated with 4 μL of a 0.5% methoxyamine–pyridine solution. The solid phase was then supplied with N-methyl-N-trimethylsilyltrifluoroacetamide for methoxylation and trimethylsilylation during derivatization and eluted with hexane. The final product was injected through a programmable temperature vaporization injector, the LVI-S250 (AiSTI SCIENCE), and the temperature was maintained at 220 °C for 0.5 min, gradually increased to 290 °C at 50 °C per minute, and held for 16 min. The samples were loaded onto a capillary column, Vf-5 ms (30 m × 0.25 mm (inner diameter) × 0.25 μm (membrane thickness); Agilent Technologies), where the temperature was maintained at 80 °C for 3 min, then increased gradually to 190 °C at 25 °C per minute, to 220 °C at 3 °C per minute, and to 310 °C at 15 °C per minute, then held for 4.6 min. After the specimens were injected at a split ratio of 20:1, each amino acid and organic acid was identified in scan mode (*m*/*z*; 70–470). All the results were evaluated for each amino acid and organic acid by normalizing the peak height of norleucine and adipic acid to 0.01 mM [18]. Alanine, valine, leucine, isoleucine, proline, glycine, serine, threonine, malic acid, aspartic acid, methionine, glutamic acid, phenylalanine, citric acid, lysine, tyrosine, and cystine were accurately quantified. Oxalic acid, malonic acid, phosphoric acid, maleic acid, succinic acid, fumaric acid, and tartaric acid could not measured because the peaks were at an inappropriate wave to scan due to low concentration or contamination.

### 2.5. Statistical Analyses

The data are presented as means ± standard deviation (SD) or frequencies of potential confounding variables. The patients were divided into two groups based on their sarcopenic risk. The differences in the continuous variables were analyzed by Student’s *t*-test and the categorical variables were analyzed using the Mann–Whitney U test and the chi-square test. Pearson’s correlation coefficient was used to analyze the correlation of each metabolite with age, HbA1c, GS, and SMI. We conducted a subgroup analysis with the same details, only for patients aged ≥ 65 years. Furthermore, we analyzed the results of the metabolome analysis and the BDHQ to determine whether each metabolite was associated with the estimated daily protein, animal protein, and plant protein intake and their ratios to each metabolite. We divided the patients into three groups according to their levels of leucine and glutamic acid, and logistic regression analysis was performed. The level of statistical significance was set at *p* < 0.05. The data were analyzed using the JMP version 14.2 software (SAS Institute Inc., Cary, NC, USA).

## 3. Results

### 3.1. Study Participants

**Figure 1:**
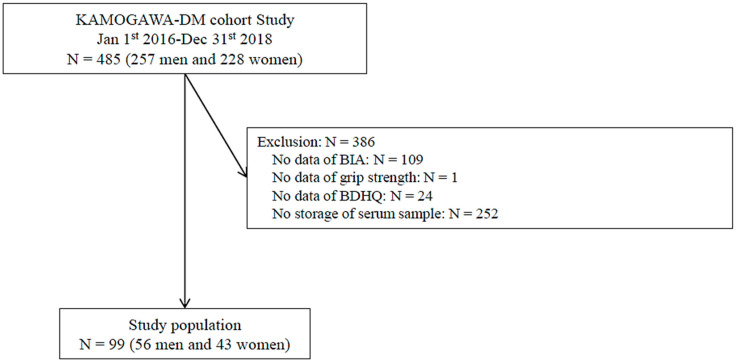
Study participants.

The inclusion criteria for the study participants are summarized in Figure 1. Out of the 99 (56 men and 43 women) participants eligible for the study, 386 were excluded due to missing BIA, GS, or serum sample data (Figure 1).

The analysis included a total of 99 patients (Figure 1), with a mean age of 63.7 ± 11.9 years and 56.6% were male. The patients had an average duration of T2D of 11.3 ± 7.7 years, a BMI of 23.9 ± 4.1 kg/m^2^, a body fat percentage of 19.0 ± 8.6%, a plasma glucose level of 143.2 ± 49.8 mg/dL, a HbA1c of 7.3 ± 1.3%, a serum Cr of 0.9 ± 0.5 mg/dL, an SMI of 7.0 ± 0.9 kg/m^2^, and a GS of 44.7 ± 12.2 kg (Table 1). Twenty-seven patients were classified as being at risk of sarcopenia, the same as the general risk, which was associated with older age (70.2 ± 2.2 years vs. 61.3 ± 1.3 years, *p* = 0.001), a longer duration of the disease (14.8 ± 1.4 years vs. 10.0 ± 0.9 years, *p* = 0.005), and a lower body mass index (21.6 ± 0.7 kg/m^2^ vs. 24.7 ± 0.5 kg/m^2^, *p* = 0.001). There was no significant correlation between the serum Cr (1.0 ± 0.1 mg/dL vs. 0.8 ± 0.1 mg/dL, *p* = 0.151), use of SGLT2 inhibitors (11.1% vs. 20.8%, *p* = 0.264) or insulin (22.2% vs. 18.1%, *p* = 0.639), and exercise habits (40.7% vs. 52.8%, *p* = 0.286). In the sub-analysis, 57 patients aged ≥ 65 years were included, with a mean age of 71.5 ± 4.7 years and 57.9% were male. The patients had an average duration of T2D of 13.0 ± 8.5 years, a BMI of 22.5 ± 3.1 kg/m^2^, a HbA1c of 7.2 ± 1.1%, an SMI of 6.8 ± 0.9 kg/m^2^, and a GS of 26.8 ± 8.4 kg. Among those aged ≥ 65 years, older age was significantly correlated with sarcopenic risk, whereas no significant correlation was observed for the duration of T2D or HbA1c.

### 3.2. Sarcopenic Risk and Plasma Metabolites

**Figure 2:**
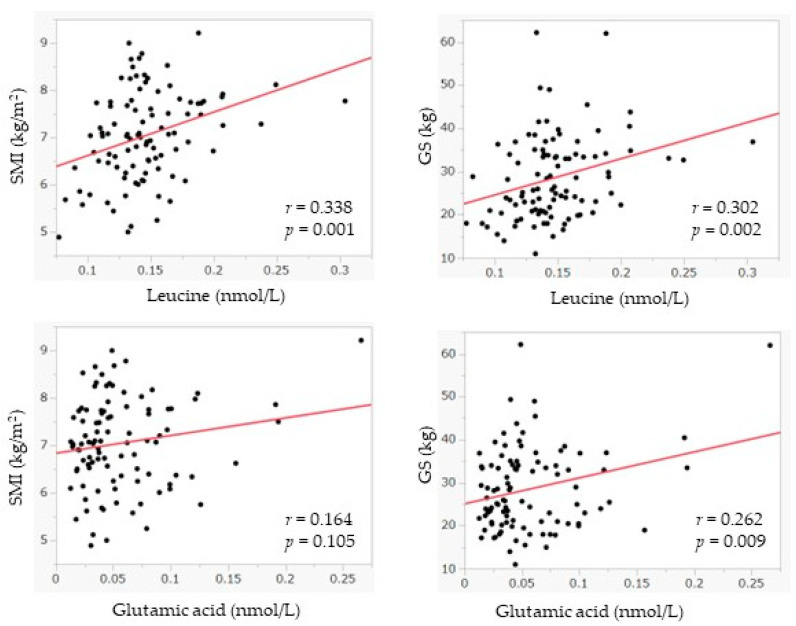
The relationship between leucine, glutamic acid, and the covariates. SMI: skeletal muscle mass index; GS: hand grip strength.

The results of the analysis indicate that the levels of serum leucine and glutamine are negatively correlated with sarcopenic risk (Table 2). The serum leucine level was 0.151 ± 0.004 nmol/L in the sarcopenic risk group and 0.135 ± 0.007 nmol/L in the non-sarcopenic risk group (*p* = 0.043), and the glutamic acid level was 0.062 ± 0.005 nmol/L in the sarcopenic risk group and 0.041 ± 0.008 nmol/L in the other group (*p* = 0.031). We also found a tendency of other amino acids levels, namely alanine (0.380 ± 0.018 nmol/L vs. 0.418 ± 0.011 nmol/L, *p* = 0.077), serine (0.115 ± 0.005 nmol/L vs. 0.126 ± 0.003 nmol/L, *p* = 0.06), aspartic acid (0.012 ± 0.002 nmol/L vs. 0.016 ± 0.001 nmol/L, *p* = 0.051), and phenylalanine (0.074 ± 0.004 nmol/L vs. 0.082 ± 0.002 nmol/L, *p* = 0.082) to be negatively correlated with sarcopenic risk, although they were not significant. However, among those aged ≥ 65 years, there were no significant relationships between all the metabolites. Additionally, the GS, which reflects muscle strength, was positively associated with the levels of leucine (*r* = 0.302, *p* = 0.002), isoleucine (*r* = 0.208, *p* = 0.039), and glutamic acid (*r* = 0.262, *p* = 0.009). SMI, which indicates muscle mass, was significantly correlated with leucine (*r* = 0.338, *p* = 0.001), and isoleucine (*r* = 0.286, *p* = 0.004). Patients with a lower SMI had lower blood metabolite concentrations for these metabolites (Table 3 and Figure 2). Regarding the association between each metabolite and age, the results show that serum levels for valine (*r* = −0.227, *p* = 0.024), leucine (*r* = −0.267, *p* = 0.008), serine (*r* = −0.24, *p* = 0.017), and glutamic acid (*r* = −0.391, *p* < 0.001) were lower in older adults, whereas cysteine was found to have higher levels in the blood of older patients (*r* = 0.317, *p* = 0.004). HbA1c was significantly correlated with glutamic acid (*r* = 0.263, *p* = 0.008), though there were no significant relationships between the other metabolites.

The logistic regression analysis indicated that those with lower levels of glutamic acid were at higher odds of sarcopenic risk after adjustment for age and HbA1c (adjusted OR 4.27, 95% CI 1.07–17.11, *p* = 0.041), although no significant associations were found for leucine (adjusted OR 1.09, 95% CI 0.34–3.51, *p* = 0.888) (Table 4). Moreover, we added serum Cr and the use of the SGLT2 inhibitor or insulin as covariates in the multivariate logistic regression analysis as additional analysis and the results did not change, which showed that patients with a lower level of glutamic acid remained at significantly higher risk of sarcopenia after adjustment for serum Cr and the SGLT2 inhibitor (adjusted OR 4.34, 95% CI 1.07–17.65, *p* = 0.04), or for serum Cr and insulin (adjusted OR 4.42, 95% CI 1.08–18.08, *p* = 0.039).

## 4. Discussion

In this study, we examined the correlation between sarcopenic risk, which is defined by a low SMI or GS, and background factors for T2D, such as age, sex, and BMI. Additionally, sarcopenic risk-related metabolites were identified through serum metabolome analysis of patients with T2D, and the association between nutritional intake and the sarcopenic risk-related metabolites was examined to determine an effective dietary therapy to prevent sarcopenia. It was found that sarcopenic risk had the same association with age as that of sarcopenia in general, and the other factors that correlated with sarcopenic risk include the duration of the T2D, weight, and BMI. However, drugs for diabetes, such as the SGLT2 inhibitor and insulin, which can affect the progression of sarcopenia or the loss of muscle mass, revealed that there was no relationship between the usage of such drugs and sarcopenic risk in the present study.

We also observed that there was a correlation between age and the levels of certain metabolites in the blood. Specifically, the levels of valine, leucine, serine, and glutamic acid were found to be lower in the elderly, while the levels of cysteine were higher. Valine and leucine are essential amino acids that are known to be more deficient in the elderly. Notably, low levels of leucine and glutamic acid were found to be significantly associated with low muscle strength (*p* = 0.002 and *p* = 0.01, respectively), and leucine was also found to be correlated with muscle mass.

This study also focused on leucine and glutamic acid as sarcopenic risk-related metabolites and their correlation with factors affecting sarcopenia. The results of the serum metabolome analysis showed that leucine and glutamic acid were correlated with sarcopenic risk, suggesting their potential as biomarkers for sarcopenia. These findings are consistent with a report based on the CATHGEN cohort [19], which identified leucine as a biomarker significantly involved in protein energy expenditure, sarcopenia, frailty, and mortality risk. Leucine is known to activate mTORC1 in muscle cells, which triggers muscle protein synthesis [20,21,22].

Glutamic acid is an amino acid that is metabolized in resting muscles [22] and provides the amino groups and ammonia necessary for glutamine and alanine synthesis, which are released after protein intake and in the post-absorptive state [22]. Previous studies have found higher levels of glutamic acid in patients with frailty due to the disrupted muscle energy metabolism associated with muscle wasting [22,23,24,25,26]. Citrulline, an end product of glutamine metabolism, is an endogenous precursor of arginine [27] that increases endothelial nitric oxide availability and vasodilation, and activates mTORC1 signaling through the hepatic catabolism of arginine and glutamine [28]. Several studies have reported an increase in citrulline serum levels with aging. The reasons for the different tendency to the previous report are as follows, serum glutamic acid is known to be lower in the presence of diabetes mellitus [29], with higher levels of HbA1c [30]. Therefore, in contrast to previous studies on non-diabetic individuals, our study of diabetic individuals indicates that lower levels of glutamic acid are associated with a higher risk of sarcopenia.

## 5. Limitations

Our study had some limitations. Firstly, the dynamics of absorption and metabolism for leucine and glutamic acid were not examined in this study, and future research will be focused on clarifying the mechanisms involved in the absorption and metabolism of these nutrients. Secondly, the study only included Japanese patients, and it is uncertain whether these results can be generalized to other nationalities. Thirdly, our study participants had a mean age of 63.7 ± 11.9 old, and a duration of diabetes of 11.3 ± 7.7 years. It has been reported that the prevalence of impaired cognitive function is increased in elderly people without exercise habits and with a longer duration of diabetes mellitus [31,32]. Then, there were some participants who were older and had a longer duration of the disease, in whom the presence of cognitive dysfunction may have influenced the current results, but there were no data on cognitive function in the present study. However, this study is significant as it is the first to investigate sarcopenia-related serum metabolites in Japanese patients with T2D by conducting metabolome analysis. Further research should investigate the relationship between each nutrient and the rate of absorption and metabolism to prevent sarcopenia.

## 6. Conclusions

In conclusion, this study demonstrated that the sarcopenic risk in Japanese patients with T2D is associated with age and the duration of the disease, as indicated by a low SMI or GS. Lower levels of glutamic acid were associated with higher odds of sarcopenic risk after being adjusted for age and HbA1c. The serum metabolome analysis revealed that leucine and glutamic acid are potential biomarkers for sarcopenia in people with T2D.

This study proves the association between serum metabolites and sarcopenic risk, which is an urgent problem for Japanese patients with T2D.

## Figures and Tables

**Table 1:** Characteristics of the study participants.

	Total	Sarcopenic Risk		*p* Value
		(−)	(+)	
N	99	72	27	-
Age, years	63.7 ± 11.9	61.3 ± 1.3	70.2 ± 2.2	0.001
Male, %	56.6	55.6	59.3	0.741
Duration of diabetes, years	11.3 ± 7.7	10.0 ± 0.9	14.8 ± 1.4	0.005
Height, cm	162.0 ± 9.3	162.3 ± 1.1	161.3 ± 1.8	0.627
Body weight, kg	62.8 ± 12.6	65.3 ± 1.4	56.1 ± 2.3	0.001
Body mass index, kg/m^2^	23.9 ± 4.1	24.7 ± 0.5	21.6 ± 0.7	0.001
Plasma glucose, mg/dL	143.2 ± 49.8	144.2 ± 5.9	140.6 ± 9.6	0.748
HbA1c, %	7.3 ± 1.3	7.4 ± 0.2	7.3 ± 0.3	0.717
Cr, mg/dL	0.9 ± 0.5	0.8 ± 0.1	1.0 ± 0.1	0.151
Skeletal muscle mass, kg	18.7 ± 4.2	19.4 ± 0.5	17.1 ± 0.8	0.017
SMI, kg/m^2^	7.0 ± 0.9	7.2 ± 0.1	6.5 ± 0.2	<0.001
GS, kg	28.5 ± 9.6	30.9 ± 1.0	22.1 ± 1.7	<0.001
Use of SGLT2 inhibitor, %	18.2	20.8	11.1	0.264
Use of insulin, %	19.2	18.1	22.2	0.639
Exercise habit (−/+)	50/49 (50.1/49.5)	34/38 (47.2/52.8)	16/11 (59.3/40.7)	0.286

Data are expressed as median ± standard deviation (SD) or number (%). SMI: skeletal muscle mass index; GS: hand grip strength. The differences in the continuous variables were evaluated using Student’s *t*-test and the categorical variables were evaluated by the Mann–Whitney U test and the chi-square test.

**Table 2:** Plasma metabolites according to the presence of sarcopenic risk.

Total	Sarcopenic Risk		*p* Value
	(−)	(+)	
N	72	27	
Alanine, nmol/L	0.418 ± 0.011	0.380 ± 0.018	0.077
Valine, nmol/L	0.272 ± 0.007	0.254 ± 0.011	0.164
Leucine, nmol/L	0.151 ± 0.004	0.135 ± 0.007	0.043
Isoleucine, nmol/L	0.077 ± 0.002	0.073 ± 0.004	0.437
Proline, nmol/L	0.184 ± 0.008	0.183 ± 0.014	0.937
Glycine, nmol/L	0.240 ± 0.007	0.262 ± 0.012	0.103
Serine, nmol/L	0.126 ± 0.003	0.115 ± 0.005	0.06
Threonine, nmol/L	0.117 ± 0.004	0.119 ± 0.006	0.773
Malic acid, nmol/L	0.011 ± 0.003	0.007 ± 0.004	0.417
Aspartic acid, nmol/L	0.016 ± 0.001	0.012 ± 0.002	0.051
Methionine, nmol/L	0.018 ± 0.001	0.018 ± 0.001	0.764
Glutamic acid, nmol/L	0.062 ± 0.005	0.041 ± 0.008	0.031
Phenylalanine, nmol/L	0.082 ± 0.002	0.074 ± 0.004	0.082
Citric acid, nmol/L	0.021 ± 0.001	0.020 ± 0.002	0.785
Lysine, nmol/L	0.120 ± 0.004	0.117 ± 0.006	0.708
Tyrosine, nmol/L	0.053 ± 0.002	0.048 ± 0.003	0.171
Cystine, nmol/L	0.011 ± 0.001	0.015 ± 0.002	0.086

Data are expressed as median ± standard deviation (SD). The differences in the continuous variables were evaluated by Student’s *t*-test.

**Table 3:** Univariate analysis: correlation between the plasma metabolite and the covariates.

Total	SMI, kg/m^2^		GS, kg		Age, years		HbA1c, %	
	γ	p	γ	p	γ	p	γ	p
Alanine, nmol/L	0.093	0.359	0.07	0.491	−0.184	0.069	0.045	0.658
Valine, nmol/L	0.19	0.059	0.183	0.07	−0.227	0.024	0.197	0.051
Leucine, nmol/L	0.338	0.001	0.302	0.002	−0.267	0.008	0.163	0.108
Isoleucine, nmol/L	0.286	0.004	0.208	0.039	−0.099	0.331	0.08	0.43
Proline, nmol/L	0.149	0.141	0.016	0.878	−0.031	0.763	−0.095	0.348
Glycine, nmol/L	−0.063	0.537	−0.193	0.056	0.112	0.272	−0.091	0.368
Serine, nmol/L	0.064	0.53	0.062	0.543	−0.24	0.017	0.17	0.093
Threonine, nmol/L	0.141	0.165	0.132	0.194	−0.037	0.713	−0.045	0.657
Malic acid, nmol/L	0.019	0.867	0.038	0.738	−0.106	0.351	−0.091	0.421
Aspartic acid, nmol/L	−0.006	0.954	0.049	0.627	−0.189	0.061	0.185	0.067
Methionine, nmol/L	0.195	0.053	0.168	0.096	0.067	0.512	−0.027	0.791
Glutamic acid, nmol/L	0.164	0.105	0.262	0.009	−0.391	<0.001	0.263	0.008
Phenylalanine, nmol/L	0.072	0.481	0.094	0.358	−0.177	0.079	0.145	0.153
Citric acid, nmol/L	0.145	0.199	0.072	0.528	0.188	0.095	−0.1437	0.226
Lysine, nmol/L	0.121	0.234	0.157	0.121	−0.12	0.236	0.11	0.281
Tyrosine, nmol/L	0.045	0.659	0.085	0.401	0.0004	0.997	0.061	0.547
Cystine, nmol/L	0.047	0.674	−0.129	0.245	0.317	0.004	−0.215	0.051

SMI: skeletal muscle mass index; GS: hand grip strength.

**Table 4:** Multiple regression analysis for the factors affecting the presence of sarcopenic risk.

Variables	Model 1 Unadjusted		Model 2 Adjusted		Model 3 Adjusted	
	OR (95% CI)	*p* Value	OR (95% CI)	*p* Value	OR (95% CI)	*p* Value
Leucine (low) (ref: high)	1.56 (0.53–4.58)	0.416	-	-	1.09 (0.34–3.51)	0.888
Glutamic acid (low) (ref: high)	5.34 (1.53–18.7)	0.019	4.27 (1.07–17.11)	0.041	-	-
Age, years	1.10 (1.04–1.17)	<0.001	1.1 (1.03–1.17)	0.005	1.1 (1.04–1.17)	0.002
HbA1c, %	0.94 (0.66–1.32)	0.711	1.13 (0.72–1.79)	0.599	1.1 (1.04–1.17)	0.913

Data represent odds ratios with 95% confidence intervals. Model 2 adjusted for age, the level of glutamic acid, and HbA1c. Model 3 adjusted for age, the level of leucine, and HbA1c.

## Data Availability

The data that support the findings in this study are available from the corresponding author upon reasonable request.
